# The Use of Human Biomonitoring to Assess Occupational Exposure to PAHs in Europe: A Comprehensive Review

**DOI:** 10.3390/toxics10080480

**Published:** 2022-08-17

**Authors:** Henriqueta Louro, Bruno Costa Gomes, Anne Thoustrup Saber, Anna Laura Iamiceli, Thomas Göen, Kate Jones, Andromachi Katsonouri, Christiana M. Neophytou, Ulla Vogel, Célia Ventura, Axel Oberemm, Radu Corneliu Duca, Mariana F. Fernandez, Nicolas Olea, Tiina Santonen, Susana Viegas, Maria João Silva

**Affiliations:** 1 Department of Human Genetics, National Institute of Health Dr. Ricardo Jorge (INSA), Av. Padre Cruz, 1649-016 Lisbon, Portugal; 2 Centre for Toxicogenomics and Human Health (ToxOmics), Nova Medical School, Universidade NOVA de Lisboa, Campo dos Mártires da Pátria, 130, 1169-056 Lisbon, Portugal; 3 National Research Centre for the Working Environment, DK-2100 Copenhagen, Denmark; 4 Istituto Superiore di Sanità (ISS), 00161 Rome, Italy; 5 IPASUM, Institute and Outpatient Clinic of Occupational, Social and Environmental Medicine, Friedrich-Alexander-Universität Erlangen-Nürnberg, 91054 Erlangen, Germany; 6 Health and Safety Executive, Buxton, Derbyshire SK17 9JN, UK; 7 Cyprus State General Laboratory, Ministry of Health, P.O. Box 28648, Nicosia 2081, Cyprus; 8 Department of Life Sciences, European University Cyprus, Nicosia 2404, Cyprus; 9 National Food Institute, Technical University of Denmark, Kemitorvet, Bygning 202, DK-2800 Kgs Lyngby, Denmark; 10 German Federal Institute for Risk Assessment, Max-Dohrn-Straße 8-10, 10589 Berlin, Germany; 11 Unit Environmental Hygiene and Human Biological Monitoring, Department of Health Protection, Laboratoire National de Santé (LNS), 1, Rue Louis Rech, 3555 Dudelange, Luxembourg; 12 Centre for Environment and Health, Department of Public Health and Primary Care, KU Leuven (University of Leuven), O&N 5b, Herestraat 49, 3000 Leuven, Belgium; 13 Centre of Biomedical Research (CIBM), University of Granada, 18016 Granada, Spain; 14 Biosanitary Research Institute of Granada (ibs.GRANADA), 18012 Granada, Spain; 15 CIBER de Epidemiología y Salud Pública (CIBERESP), 28029 Madrid, Spain; 16 Finnish Institute of Occupational Health, 00250 Helsinki, Finland; 17 Public Health Research Centre, NOVA National School of Public Health, Universidade NOVA de Lisboa, 1600-560 Lisbon, Portugal; 18 Comprehensive Health Research Center (CHRC), 1169-056 Lisbon, Portugal

**Keywords:** polycyclic aromatic hydrocarbons, occupational exposure, human biomonitoring, exposure biomarker, effect biomarker

## Abstract

Polycyclic aromatic hydrocarbons (PAHs) are among the chemicals with proven impact on workers’ health. The use of human biomonitoring (HBM) to assess occupational exposure to PAHs has become more common in recent years, but the data generated need an overall view to make them more usable by regulators and policymakers. This comprehensive review, developed under the Human Biomonitoring for Europe (HBM4EU) Initiative, was based on the literature available from 2008–2022, aiming to present and discuss the information on occupational exposure to PAHs, in order to identify the strengths and limitations of exposure and effect biomarkers and the knowledge needs for regulation in the workplace. The most frequently used exposure biomarker is urinary 1-hydroxypyrene (1-OH-PYR), a metabolite of pyrene. As effect biomarkers, those based on the measurement of oxidative stress (urinary 8-oxo-dG adducts) and genotoxicity (blood DNA strand-breaks) are the most common. Overall, a need to advance new harmonized approaches both in data and sample collection and in the use of appropriate biomarkers in occupational studies to obtain reliable and comparable data on PAH exposure in different industrial sectors, was noted. Moreover, the use of effect biomarkers can assist to identify work environments or activities of high risk, thus enabling preventive risk mitigation and management measures.

## 1. Introduction

Polycyclic aromatic hydrocarbons (PAHs) are mostly formed during incomplete combustion of organic materials such as coal, coal-derived products, oil, and its by-products, natural gas, and wood [1]. Anthropogenic activities using these organic materials generate environmental air pollution, primarily in big cities where the increasing population requires a growth in the use of hydrocarbon-based fuels for transportation, heating systems, and electric power generation. To a lesser extent, PAHs are also produced by lifestyle habits (e.g., tobacco smoke and consumption of grilled food) and fires (e.g., wildland and structure fires). Although exposure to PAHs in the general population may raise some interest, occupational exposure raises the major concern because workers are frequently exposed to far higher concentrations and for longer periods of their lives [1,2,3]. 

Occupational exposure to PAHs occurs mainly in industrial sectors such as coking, impregnation processes with tar, creosote, and bitumen, aluminum production and smelting, asphalt industry, mining, metal working, oil refining, and also in non-industrial sectors such as firefighting [3]. PAHs can be absorbed by inhalation, ingestion, and via the skin [4]. Inhalation, followed by dermal contact, are the primary routes of workers exposure [5,6]. Absorption through inhalation is very quick and efficient, and upon exposure to pyrene, a pulmonary retention of 61% has been estimated [7]. High and efficient absorption of PAHs through human skin has been also demonstrated [8,9,10]. Once absorbed, PAHs are rapidly transported via the bloodstream and lymph to every tissue. Because of their lipophilic nature, PAHs can accumulate in adipose tissue, which may act as a depot from which the PAHs are released over time [11,12]. 

PAHs are metabolized primarily in the liver and kidney, through phase 1 and 2 reactions, such as oxidative, hydroxylation, reductive, and conjugative processes [13,14,15]. The first step of metabolism is always an epoxidation of the aromatic system by cytochrome P450 isoenzymes (CYP) [14]. The major transformation of any epoxide is the epoxide hydrolase catalyzed hydroxylation which results in a di-hydrodiol product. Besides hydroxylation, the epoxide can also undergo phase 2 reactions, e.g., the glutathione-S-transferase controlled addition to glutathione. These reactions result in the corresponding conjugates, which can easily be eliminated via urine ). On the other hand, epoxides can react spontaneously with nucleophilic reaction sites of macromolecules, like proteins and ribonucleic acids [14].

The most prominent PAHs metabolites are the mono-hydroxylated metabolites, which result from isomerization of the epoxides, and are potential target molecules for human biomonitoring (HBM) of PAHs exposure. The formation of these metabolites have been demonstrated for naphthalene (NAP), phenanthrene, fluorene, chrysene, pyrene, and benzo[a]pyrene (BaP) [16,17,18,19]. Also relevant are aldo-keto reductase (AKR) pathways. For example, 1,2-dihydroxynaphthalene, which results from the AKR pathways, is by far the main urinary metabolite of naphthalene [20]. This urinary metabolite has shown a strong correlation with environmental naphthalene exposure, which supports its use as a biomarker for the human biomonitoring of naphthalene exposure as an alternative for more commonly used 1- and 2-naphthol [20]. Pyrene is frequently found in PAH mixtures and its urinary metabolite, 1-hydroxypyrene (1-OH-PYR), has been commonly used as an indicator of exposure to PAHs [21,22]. Overall, many PAHs metabolites with potential for use as exposure biomarkers have been already identified [16,17,18,19], as follows: 1-hydroxynaphthalene (1-OH-NAP), 2-hydroxynaphthalene (2-OH-NAP), 1,2-dihydroxynaphthalene (1,2-OH-NAP), 2-hydroxyfluorene (2-OH-FLU), 3-hydroxyfluorene (3-OH-FLU), 9-hydroxyfluorene (9-OH-FLU), 1-hydroxyphenanthrene (1-OH-PHE), 2-hydroxyphenanthrene (2-OH-PHE), 3-hydroxyphenanthrene (3-OH-PHE), 4-hydroxyphenanthrene (4-OH-PHE), 1,2-dihydroxyphenanthrene (1,2-OH-PHE), 3,4-dihydroxyphenanthrene (3,4-OH-PHE), 9,10-dihydroxyphenanthrene (9,10-OH-PHE), 6-hydroxychrysene (6-OH-CHR), 1-hydroxypyrene (1-OH-PYR), 1,6-dihydroxypyrene (1,6-OH-PYR), 1,8-dihydroxypyrene (1,8-OH-PYR), 3-hydroxybenzo[a]pyrene (3-OH-BaP), 1-hydroxyacenaphtene (1-OH-ACE).

The elimination kinetics after inhalation, oral, and dermal absorption indicates a possible systemic accumulation of PAHs during consecutive exposure, which has been confirmed in several studies [23,24,25,26]. Data available concerning the kinetics of some PAHs metabolites are summarized in Supplementary Table S1. 

PAHs are well known to be hazardous to human health. The main effect of PAH exposure is cancer, namely lung and skin cancer [27], and, to a lesser extent, non-cancer effects including cardiovascular [28,29,30] and dermal toxicity (reviewed in [31]).. Many PAHs are classified as probable or possible carcinogens according to the International Agency for Research on Cancer [32]. BaP is classified as carcinogenic (IARC group 1), as well as some PAH mixtures, for example, soot [33], diesel engine exhaust [34], and outdoor air pollution [35]. PAHs can also act as endocrine disruptors [36]. Due to their carcinogenicity, the presence of PAHs in several consumer products is restricted in the European Union. Some PAHs congeners are included in the List of substances of very high concern (SVHC) for possible future Authorization in accordance with Article 59 (10) of the REACH Regulation [37]. Carcinogenic PAH mixtures have been also included in EU directive 2004/37/EC on the protection of workers from the risks related to exposure to carcinogens or mutagens at work, with a skin notation due to the possibility of significant uptake through the skin. However, no binding OEL has been adopted for PAHs or BaP at the EU level. 

In order to warrant safe exposure levels, monitoring PAHs is usually performed by means of stationary or personal air-sampling at the workplace, which allows characterizing external exposure and inferring workers’ exposure via inhalation. However, biomarkers of PAHs exposure measured in urine or blood, allow the assessment of the aggregated internal exposure level achieved by inhalation, dermal and gastrointestinal routes. Most of the air monitoring methods available for measuring workplace exposure to PAHs allow the measurement of the 16 priority PAHs (e.g., NIOSH method 5528; https://www.cdc.gov/niosh/nmam/pdf/5528.pdf (accessed on 27 June 2022)). There are no EU-wide biological limit values (BLV) for PAHs. The Scientific Committee on Occupational Exposure Limits (SCOEL) concluded in its opinion published in 2016 that 1-OH-PYR represents the main metabolite of pyrene in mammals and has become accepted as a sensitive and specific marker of PAHs exposure, for which reliable and robust analytical methods are described [28]. However, since PAHs were considered non-threshold carcinogens, no health-based BLV were given, but a biomonitoring guidance value (BGV) of 0.5 µg 1-OH-PYR per g creatinine (determined after conjugate hydrolysis) was proposed to identify occupational exposure from background general population exposure level.

In this work, we describe a systematic review of the literature published between 2008 and 2022 on occupational exposure to PAHs in Europe, developed under the H2020 European Human Biomonitoring Initiative (HBM4EU). An overview of the use of human biomonitoring data is presented, including the HBM parameters applied in occupational studies on PAHs exposure and effects, PAHs metabolites levels reported, and the occupational settings where workers incur in the greatest risk. The knowledge gaps in HBM studies concerning PAHs are identified and the most common and suitable effect biomarkers to assess early biological effects are also discussed.

## 2. Materials and Methods

### 2.1. Search Strategy for Papers Concerning HBM of Occupational PAHs Exposure

A systematic literature search on the use of HBM in occupational exposure to PAHs studies was conducted. Papers published between 2008 and March 2022, from different sources, were used: PubMed, Web of Science, and Scopus and were evaluated according to the PRISMA methodology [38]. An outline of the different phases of the search, together with the number of papers identified, can be found in the results section. 

At the first stage, broad search terms and filters were defined to identify occupational studies that used human biomonitoring to evaluate exposure to PAHs (Table 1).

After the initial removal of duplicates by one member of the team, all retrieved titles and abstracts were independently screened, in parallel by four members of the team, using previously established inclusion/exclusion criteria (Table 2). Articles that did not meet the inclusion criteria were excluded from further analysis.

In this search, occupational HBM studies performed in different activity sectors where exposure to PAHs can occur were included. All types of studies were considered (cross-sectional, cohort, and case-control studies), and no minimum limit on the number of individuals included in the study was set. Non-epidemiological studies, e.g., studies on analytical methods and new biomarkers, were excluded.

### 2.2. Quality Scoring of the Articles Retrieved

The quality of each study in regard to our purpose was assessed using a modification of the LaKind scoring method [39]. Briefly, the scoring method was based on quality criteria for three fundamental areas of epidemiological studies that include biological measurements of short-lived chemicals: (1) biomarker selection and measurement; (2) study design and execution; and (3) general epidemiological study design considerations. In Table 3, the components analyzed within these areas are shown. A value from 1 to 3 was assigned to each parameter, where 1 is the best in the quality criteria score.

One of the aims of the H2020 European Human Biomonitoring Initiative (HBM4EU) was the development of the field of effect biomarkers to implement them in a more systematic and harmonized way in large-scale European HBM studies. The implementation of effect biomarkers complements exposure data with mechanistically based biomarkers of early and late adverse effects. Therefore, information about the effect biomarkers used and results obtained was also collected from the articles retrieved, following the literature search conducted according to criteria presented in Table 1 and Table 2.

## 3. Results

### 3.1. Literature Search and LaKind Scoring

**Figure 1:**
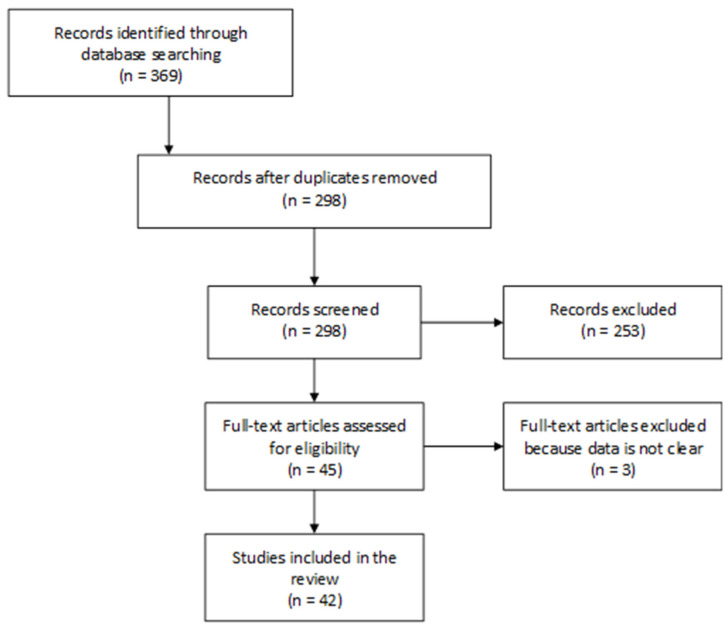
Diagram showing the different phases in the HBM studies selection in this review.

The search strategy for exposure assessment in HBM studies led to the identification of 369 articles in total, including 227 from PubMed, 53 from Web of Science, and 89 from Scopus. Of these 369 articles, 42 were eligible to be included in this review (Figure 1).

The results of the LaKind scoring that was applied to the 42 studies selected for review (Supplementary Table S2) showed that the highest score (lowest quality) obtained was 18 [40] and the lowest score (highest quality) was 10 [41]. This scoring system is not a judgment of the scientific quality of the paper, but it is rather an assessment of to which degree it answers the questions raised in this review. Many studies scored poorly on quality assurance parameters; this is not to say that studies were poorly conducted but highlights that still today many studies do not adequately report their use of quality control and quality assurance procedures. Although the poorer scoring papers were older, indicating an improvement in reporting in recent years, there were still high-quality data papers across the date range.

### 3.2. Exposure Biomarkers and Occupational Exposure (Limit) Values

Among the 42 articles eligible in this study, the main occupational settings focused were firefighting, coke oven, asphalt/bitumen/rode paving, metallurgic and electrode industries, aluminum production, waste incineration, restaurant workers, policemen, drivers, and nurses involved in the topical application of coal tar ointment. Additionally, air force personnel, green spaces workers and roofers, navy workers as well as health care workers were also reported (Table 4).

Table 4 summarizes the biomarkers of exposure to PAHs as reported in the occupational studies included in the present review. PAHs include a mixture of multiple substances and thereby typically one or a few representative PAHs metabolites have been used as urinary markers in biomonitoring studies. The most frequently used biomarkers were: 1-OH-PYR (32 out of 42 studies) followed by 3-hydroxyBaP (3-OH-BaP) (7 out of 42 studies). These two exposure biomarkers were used in the study showing the best score by the Lakind scoring [41]. In addition, other biomarkers have been used, namely, 1-hydroxynaphthalene (i.e., 1-naphthol, 1-OH-NAP); 2-hydroxynaphthalene (2-naphthol, 2-OH-NAP); 2-hydroxyfluorene (2-OH-FLU); 9-hydroxyfluorene (9-OH-FLU); 1-hydroxyphenanthrene (1-OH-PHE); 2-hydroxyphenanthrene (2-OH-PHE); 3-hydroxyphenanthrene (3-OH-PHE); 4-hydroxyphenanthrene (4-OH-PHE); 9-hydroxyphenanthrene (9-OH-PHE) and 6-hydroxychrysene (6-OH-CHR) (Table 4).

**Figure 2:**
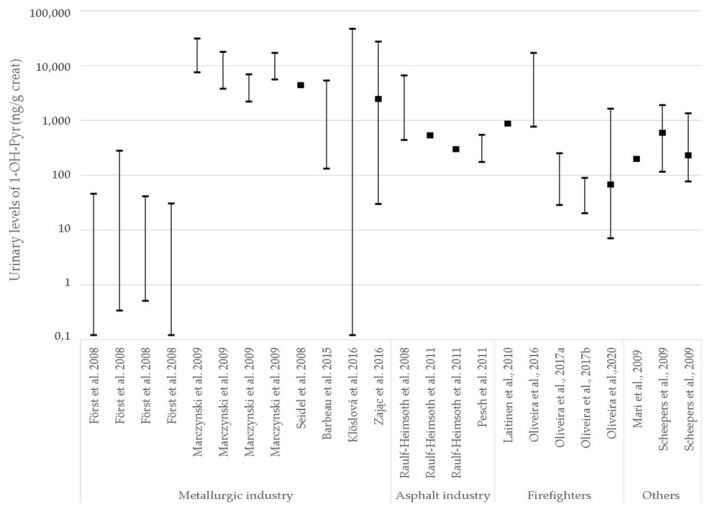
Urinary levels distribution of 1-OH-PYR across several industries in the literature. Creat: creatinine. Minimum and maximum values are shown together with median/mean (black square) whenever available. The y-axis is depicted in logarithmic scale.

Regarding external exposure, the exposure levels of individual PAHs in air vary according to the industry sector. Not all studies eligible for this review analyzed the potential associations between PAHs detected in air and the biomarkers of exposure used. The most detected PAHs in French metallurgic industries were Pyr, BaP, Nap, Flu, and Phe [43,44]. In those studies, 1-OH-PYR correlated with gaseous Pyr and particle Pyr, whereas 3-OH-BaP did not correlate with the measured BaP air levels [43,44]. In another study, the same authors reported a weak correlation of 1-OH-NAP, 2-OH-NAP, 1-OH-PHE and 3-OH-PHE with workplace air levels of parent PAHs and a strong correlation of 2-OH-FLU, 3-OH-FLU, and 2-OH-PHE with air levels of their parents’ PAHs [44]. Using the same air markers, three studies have shown a correlation between PAHs levels in air samples and metabolites in urine [45,47,48]. In fact, Förster et al. [45] also reported correlations between urinary levels of 3-OH-BaP and ∑OH-PHEs and 1-OH-PYR, proving that in metallurgic industries, these three hydroxylated PAHs can be surrogates of each other. Regarding asphalt industries, the majority of studies that measured PAHs in the air and exposure biomarkers were performed in Germany [49,50,53]. All studies measured the 16 US EPA PAHs in air and as biomarkers of exposure, the authors used 1-OH-PYR, ∑OH-PHE and ∑OH-NAP. The authors have not shown a correlation between air concentrations and urinary biomarkers since post-shift urinary PAHs metabolites concentrations did not reflect the different exposure levels. In other industries, such as coke oven [58] and aluminum production [46] the PAHs markers used for air monitoring were ∑PAHs and BaP. Zając et al. reported a correlation between 1-OH-PYR, ∑PAHs, and BaP, showing that 1-OH-PYR might be a surrogate for BaP and PAHs in general [58]. Furthermore, 1-OH-PYR levels in urine showed a wide distribution across different studies (Figure 2) reflecting wide ranges of exposure, either related to air or to other exposure routes, such as dermal exposure.

In this sense, in two German studies of workers in different industries with high PAHs exposures, exposure was assessed both by measuring air PAHs concentrations and PAHs metabolites in urine [47,52]. The authors discuss that the observed lack of association between air concentrations of PAHs and the urinary PAHs metabolite levels indicates that other routes of exposure, in addition to inhalation of PAHs, are of importance [47].

The above studies show that PAHs uptake through the dermal route may be significant, thus favoring the use of biomonitoring data for the assessment of occupational PAH exposure in addition to air measurements.

Monohydroxylated PAH metabolites in urine, mostly 1-OH-PYR and also the five hydroxyphenanthrenes (1-, 2-, 3-, 4-, and 9-OH-PHE), have been widely accepted as reliable biomarkers for internal exposure to PAHs [81]. Among the studies reviewed, they were extensively used for the estimation of PAHs exposure in different work sectors, e.g., steel production and coke plants [45,47,48,58], waste incinerators [63,64], asphalt workers [49,53], policemen [73], firefighters [40,65], and dermatology nurses [77].

### 3.3. Effect Biomarkers 

Among the 42 studies selected for the present review, eleven studies reported the use of effect biomarkers in occupational exposure to PAHs [6,47,50,51,52,69,70,73,74,75,76,79]. The reported effect biomarkers primarily included markers of genotoxicity/oxidative stress (nine studies) and cardiovascular effects (six studies). An overview of these studies is presented in Table 5 and Table 6. 

Nine of the studies focused on DNA damage: (1) oxidative damage of DNA or RNA, (2) DNA strand breaks, and/or (3) micronuclei (Table 5). The most frequent biomarker of genotoxic/oxidative damage used was DNA strand breaks (five studies), followed by 8-oxodG levels in either blood or urine (3 studies) and formamidopyrimidine DNA glycosylase (Fpg)-sensitive sites in PBMC (2 studies), respectively. In addition, 8-oxoGuo, 8-OHdG, and micronuclei were analyzed in a single study each. A strong correlation between urinary OH-PYR, dermal PAH exposure, and DNA strand breaks in the blood was found in the study of recruits during training as firefighters [6].

The 8-oxodG levels were increased in bus drivers (n = 50) compared to controls (n = 50) in a Czech cross-sectional study. Neither personal nor stationary air concentrations of PAHs, B(a)P or VOC correlated with the 8-oxodG levels. Instead, the 8-oxodG levels correlated with PM2.5 and PM10 concentrations [74]. Increased levels of 8-oxo-dG and DNA strand breaks compared to the control group were observed in coke oven (n = 37), refractory (n = 96), graphite electrode (n = 26), and converter workers (n = 12) as compared to a control group consisting of construction workers (n = 48) in a German cross-sectional study [47]. The highest levels of DNA damage were observed for the graphite-electrode production workers. DNA damage levels were not associated with PAHs exposure measured as air PAHs concentrations or urinary PAHs metabolite levels [47]. 

Furthermore, increased levels of 8-oxo-dGua adducts and DNA strand breaks were found in bitumen-exposed construction workers (both pre- and post-shift). The level of 8-oxo-Gua was increased post- compared to pre-shift, whereas the level of DNA strand breaks was decreased post- compared to pre-shift. No difference between the levels of (+)-anti-BPDE-DNA adducts in exposed and control samples was observed. No association between exposure to PAHs (measured as urinary metabolites of PAHs or air concentration of PAH) and DNA damage in blood cells was found [52]. Moreover, increased levels of 8-OHdG in second-hand tobacco smoke-exposed workers from Portuguese restaurants were observed compared to non-exposed workers, regardless of smoking status [79]. Proteomics analysis showed nine differentially expressed proteins in plasma of second-hand tobacco smoke-exposed non-smokers. Among these, two acute phase proteins inter-α-trypsin inhibitor heavy chain 4 (ITIH4) and ceruloplasmin (CP) were the most differentially expressed proteins [79]. Genotoxicity effect biomarkers were also used and no clear association between occupational exposure to second-hand smoke and the induction of genotoxicity was observed, although the leukocytes from non-smoking second-hand tobacco smoke-exposed individuals displayed lower DNA damage levels in response to an ex vivo challenge, in comparison to those from non-exposed workers, suggesting a possible adaptive response [82].

The biomarkers of genotoxicity used in the selected studies are on the list of identified effect biomarkers for BaP identified by IARC [33]. In addition to DNA damage (measured by the comet assay) and 8-oxo-deoxyguanosine formation, IARC also lists chromosomal aberrations and sister chromatid exchange as biomarkers of genotoxicity.

Four out of the described studies, indicated increased ROS production and genotoxicity, in workers exposed to PAHs compared to controls, but did not find a correlation between PAHs exposure and genotoxicity [47,52,74,79]. 

Six studies also included biomarkers of cardiovascular effects (Table 6), three of them analyzing different acute-phase proteins (SAA, CRP, ITIH4, and CP) and/or inflammatory markers (Il-6, IL-8, V-CAM, and I-CAM) without noticing significant increases in the exposed population [6,69,79]. One study found that 1-OH-PYR was negatively associated with systolic and diastolic BP [73], while another study found decreased microvascular function and altered heart rate variability indicating an imbalance in the autonomic activation of the heart [69].

## 4. Discussion

### 4.1. Strengths and Limitations of the Strategies Used for HBM of PAHs

Choosing the most appropriate biomarkers to realistically describe the exposure to PAH mixture in each occupational setting is of most importance since health effects depend not only on the PAHs levels but also on the mixture composition. However, this will also require the development of risk assessment models for the interpretation of the impact of varying compositions on the risks of PAH mixtures since the current dose responses for PAH mixtures are based on BaP as an indicator of exposure.

This literature review revealed that most of the selected studies use BaP as a marker for general airborne PAH exposure, justified mainly for practical reasons [28]. However, for some occupational settings, BaP may not be the appropriate indicator of exposure to these compounds. In addition, there are occupational scenarios where airborne PAH exposure is dominated by PAHs of lower ring number, such as naphthalene, anthracene, and phenanthrene [83]. It would therefore be preferable to monitor a broad spectrum of PAH components or at least total PAH exposure, as the relative abundance of other PAHs with adverse effects on human health may vary depending on the occupational setting. When the content of the PAH mixture is unknown, exposure to the 16 priority PAHs should be assessed.

The 1-OH-PYR, the pyrene metabolite, was the most widely used exposure biomarker in the reviewed occupational studies, although it is only an indirect marker of exposure to carcinogenic PAHs, including BaP. Nevertheless, the relative content of pyrene compared to other PAHs is well-studied and fairly constant in air samples from working environments [81]. Moreover, a good correlation between airborne BaP and post-shift urine levels of 1-OH-PYR has been reported (if samples from workers using respiratory protection or with significant dermal exposure were excluded) [84]. The combination of highly sensitive and relatively cheap detection methods (e.g., urinary 1-OH-PYR) and evidence of linear correlation with airborne exposure suggests 1-OH-PYR as a robust biological exposure biomarker [81]. However, the use of PYR and PHE metabolites to assess the carcinogenic risk associated with occupational exposure to PAHs has been questioned [32]. Recently, new biomarkers have been implemented that can provide information on exposure to PAHs of greater toxicological relevance than pyrene, such as 3-OH-BaP, the main urinary metabolite of BaP [85]. Several aspects, however, may negatively affect the routine use of 3-OH-BaP as a reliable biomarker of occupational PAHs exposure. The predominance of the fecal over the urinary excretion pathway for BaP metabolite contributes to very low urinary 3-OH-BaP levels, which requires particularly highly sensitive analytical procedures. Therefore, most studies using 3-OH-BaP are mainly exposure scenarios entailing high occupational exposure, such as those observed among metallurgy workers [41,42] and firefighters [67]. A very recent work analyzed the consistency between air and biological monitoring for PAHs exposure and cancer risk of workers, suggesting an overall agreement between airborne PAH levels and urinary biomarker concentrations [86]. The use of urinary 1-OH-PYR seemed to be more protective than those of urinary 3-OH-BaP and air BaP [86].

The study design of biomonitoring studies may greatly impact the statistical power to detect the effects of exposure. Longitudinal and cross-shift studies, where the volunteers act as their own controls, eliminate the contribution from inter-individual variation as compared to cross-sectional studies. Furthermore, using healthy volunteers or previously unexposed persons may also improve the ability to detect the effects of occupational exposure to PAHs, because occupationally exposed persons (such as firefighters) may have increased pre-shift levels of PAHs due to previous exposures [6]. Needless to say, the number of persons recruited in the studies is an extremely important determinant for the statistical power of a study. Pooled studies across Europe have greatly increased the statistical power as seen in the studies of occupational exposure to hexavalent chrome [87]. In the HBM4EU studies, the study design and detection methods were harmonized to ensure comparability of the biomonitoring data. For PAHs detection, several different methods for quantification were used in the studies reported in this review, and it is not clear whether these are entirely comparable.

The exposure of humans to NAP has also become an environmental and occupational health concern since international agencies classify NAP as a possible human carcinogen [33]. Monohydroxylated metabolites (1- and 2-OH-NAP) have been widely used as biomarkers for the assessment of occupational exposure to NAP at different workplaces [44,53,55,67]. However, the high background levels found in non-occupationally exposed populations and in non-smoker urine samples give rise to doubts about their specificity as occupational biomarkers; 1-OH-NAP is also not specific to naphthalene exposure as it can be formed from carbaryl exposure [88]. Klotz et al., evaluated the suitability of several NAP metabolites, including 1- and 2-OH-NAP, for their application in biomonitoring studies [60]. The dihydroxylated metabolite 1,2-OH-NAP was found to be the most sensitive and 1-naphthylmercapturic acid, the most specific biomarker for biological monitoring of occupational exposure to NAP. However, to date, the latter has not been widely used [60].

The use of effect biomarkers in biomonitoring studies, including occupational studies, is useful for the early identification of subclinical diseases. More importantly, the establishment of relationships between PAHs exposure and biomarkers of effect can be used for risk assessment, as well as for the establishment of exposure limits, both in the occupational and general environment [5]. This review showed that, for genotoxicity, chromosomal aberrations, sister chromatid exchange, DNA damage measured by the comet assay, 8-oxo-deoxyguanosine formation, and micronuclei are established human effect biomarkers in relation to exposure to BaP-containing mixtures, in line with IARC view [32,87]. A review evaluated the effect of PAHs exposure on micronuclei induction in 34 occupational studies showing that the micronucleus assay may be a sensitive biomarker of PAHs exposure in contaminated workplaces [89]. Furthermore, experimental studies with rodents exposed to BaP or anti-BaP-7,8-diol-9,10-oxide support this association [33]. 

Among the limitations of the search performed, it should be noted that the selected articles included mainly biomarkers of genotoxicity, oxidative stress, and cardiovascular effects, ignoring other effect biomarkers of interest (e.g., proteomic, metabolic, or endocrine disrupting markers), to evaluate the effect of PAHs [90,91,92].

Assessing exposure to PAHs by measuring only airborne concentrations is one of the main limitations found, as other uptake pathways can considerably contribute to internal PAHs exposure to these pollutants [79]. Thus, the importance of the dermal route of PAHs uptake has again been shown in three recent studies of individuals engaged in firefighting [70], where a statistically significant correlation between dermal PAHs exposure and urinary PAHs metabolites was observed [6,71,93]. These studies also showed that firefighting was associated with increased levels of DNA strand breaks in blood cells and increased urinary mutagenicity, compared to controls [6,93]. Moreover, the study by Andersen et al. reported statistically significant correlations between urinary PAHs metabolite levels and DNA damage levels in blood cells, and between dermal exposure to pyrene and ƩPAHs and DNA damage levels [6], suggesting that dermal PAHs exposure contributed to the observed genotoxicity. Genotoxic effects have also been demonstrated following dermal exposure to PAHs in hairless mice [94]. 

Five of the 11 studies selected with effect biomarkers, focused on biomarkers of cardiovascular disease, specifically the association between PAHs exposure and blood pressure [29,72]. Furthermore, biomarkers of oxidative stress and acute phase response in restaurant workers exposed to second-hand smoke suggested that acute phase proteins, CP and ITIH4, could be candidate biomarkers to monitor second-hand smoke exposure of hospitality workers [79]. In fact, the acute phase response has already been proposed as a possible mechanism of particle-induced cardiovascular disease [95,96,97]. Serum levels of the acute phase proteins C-reactive protein (CRP) and serum amyloid A (SAA) have also been associated with an increased risk of cardiovascular disease in prospective epidemiological studies [98], just as SAA has also been shown to promote atherosclerotic plaque progression, suggesting a causal role in this disease [99].

### 4.2. HBM Added Value, Gaps and Needs for New Data

A major advantage of HBM data is that it provides an integrated overview of the body burden to selected chemicals and serves as a good estimate of aggregate exposure. In addition, inter-individual variation in uptake, metabolism, and excretion is taken into account. Where respiratory protective equipment (RPE) is worn (e.g., coke ovens), biomonitoring can give an insight into the efficacy of the RPE used and how it is being used. Furthermore, the importance of using biomonitoring tools in occupational exposures to PAHs is justified because these substances are known to be absorbed through the skin and the levels of urinary 1-OH-PYR are particularly high in occupationally exposed populations where dermal exposure is likely [10,100]. However, the dermal exposure route seems to imply a later peak of urinary excretion when compared with the respiratory route with possible continued absorption due to the continuous contact with contaminated clothes and expected penetrating of skin layers and deposit effect [101]. Therefore, and to allow for a correct interpretation of biomonitoring data, a detailed description of the tasks carried out, the duration of tasks, the possible dermal contamination, and the personal protective equipment worn is also needed [100,102]. 

However, HBM studies should be standardized allowing a more accurate comparison of the PAHs levels across studies and also a more homogeneous publication of the data. This, in turn, requires the use of individual HBM data, accompanied by ancillary information that would shed light on the mechanistic link between exposure dynamics and observed HBM data. This will allow a much better comparison and allow derivation of reference values-based regulations. In fact, at the moment, many studies in Europe use different approaches that in many cases could not be directly comparable.

It seems relevant when developing new occupational studies with harmonized approaches, to apply a set of exposure biomarkers that can be related to the PAHs mixture present in the setting being studied, rather than focusing only on BaP, especially since there is evidence that PAHs exposure is associated not only with cancer risk but also additional health outcomes such as cardiovascular disease. Within HBM4EU, the exposure biomarkers selected for general population studies have been discussed and the most frequently detected monohydroxy-PAHs, 1- and 2-OH-NAP, 1-OH-PYR, 2-, 3-, and 9-OH-FLU and 1-, 2-, 3- 4, and 9-OH-PHE, were selected [103]. A number of European labs underwent a quality assurance process and were qualified for the analysis of these metabolites [103]. 

Future development of a proposal for a biomonitoring strategy of PAHs to be used in occupational settings, including a specific flowchart considering the exposure scenarios specificity, can be defined based on the information presented here. The approach followed in previous papers for other chemicals of concern [104,105,106] is a good example to consider for PAHs. In addition, effect biomarkers, e.g., indicators of genotoxic and carcinogenic effects or of cardiovascular effects may help early identifying biological alterations and thereby a risk of disease that can be prevented through risk management measures before disease development.

## Figures and Tables

**Table 1:** Search terms used in the different literature databases.

Source	Keywords	Filters
PubMed	PAHs; polycyclic aromatic hydrocarbons; environmental monitoring; biomonitoring; occupational exposure	Full text; January 2008 to March 2022; English; human
Web of Science	PAHs; polycyclic aromatic hydrocarbons; biomonitoring; occupational exposure	All databases; January 2008 to March 2022; English
Scopus	polycyclic aromatic hydrocarbons; biomonitoring; occupational exposure	All databases; January 2008 to March 2022; English

**Table 2:** Inclusion and exclusion criteria for the literature search.

Criteria	
Inclusion criteria	Publication date between January 2008 and March 2022 * English language Report exposure from European workplaces Studies with quantitative data concerning occupational exposure to PAHs. This includes data obtained from HBM and from air monitoring in different exposure scenarios
Exclusion criteria	Published before 2008 Non-English language Studies reporting exposure outside of Europe Studies reporting exposure of general population Grey literature/not published in a peer reviewed journal Dissertations/theses Proceedings Only abstract available Reviews containing only published data

* The search was performed in September 2018, including articles ahead of print, and was updated in March 2022.

**Table 3:** Adaptation of LaKind scoring method to HBM studies concerning PAHs.

Assessment Component	TIER 1	TIER 2	TIER 3
Study participants	>20 occupationally exposed individuals	5–20 occupationally exposed individuals	Any other study (<5 occupationally exposed individuals)
Chemicals under investigation	8 carcinogenic PAHs in entry 50 of Annex XVII to REACH (PAH8): BaP, benzo[e]pyrene, benzo[a]anthracene, chrysene, benzo[b]fluoranthene, benzo[j]fluoranthene, benzo[k]fluoranthene, and dibenzo[a,h]anthracene.	Other than PAH8 or fewer than the 8 carcinogenic PAHs in entry 50 of Annex XVII to REACH	Other contaminants that result from the same process where PAHs can be produced.
Exposure biomarker and matrix	Biomarker(s) in a specified matrix has accurate and precise quantitative relationship with external exposure, internal dose, or target dose. E.g., BaP-specific metabolites, in particular 3-hydroxyBaP (3-OH-BaP).	Evidence exists for a relationship between biomarker in a specified matrix and external exposure, internal dose, or target dose but limited application. E.g., 1-OH-PYR, metabolite of pyrene-indirect marker of exposure to PAH mixtures that include BaP; glucuronide of 1-OH-PYR; OH-PHEs.	Biomarker in a specified matrix is a poor surrogate (low accuracy and precision) for exposure/dose. E.g., carboxyhemoglobin or volatile organic compounds (VOC) in exhaled air.
Biomarker specificity	Biomarker is at least one of the PAH8 or a specific metabolite. E.g., BaP and/or 3-OH-BaP	Biomarker is one PAH or a specific metabolite not included in PAH8. E.g., pyrene and/or 1-OH-PYR	Biomarker is derived from contaminants that result from the same process where PAHs can be produced.
Technique	Analytical methods that provide unambiguous identification and quantitation of the biomarker at the required sensitivity (e.g., HPLC-FD, GC-HRMS, LC-MS/MS).	Other analytical methods that provide quantitative but potentially ambiguous identification of the biomarker (e.g., HPLC).	Analytical methods that only allows for detection of the biomarkers but is not able to quantify.
Method characteristics	Acceptable LOD; Samples with a known history and documented stability data; samples are contamination-free	Stability not specifically assessed, but samples were stored appropriately and analyzed promptly.	LOD above current state-of–the-art; specific reason to query stability; known contamination issues
Quality assurance (QA)	Study has used external QA where appropriate	Some QA used (note details)	No QA
Sampling strategy and matrix adjustment	-	Study includes results for adjusted concentrations if adjustment is needed;	

LOD: level of detection; QA: quality assurance.

**Table 4:** Summary of exposure biomarkers identified in occupational exposure to PAHs as reported in the studies of interest to the present review.

Occupational Sector	Study Design and Country	Biomarkers of Exposure and Effect	External Exposure Monitoring	Comments	Ref.
Metallurgic Industry	129 male subjects (18–65 years old) working in anode, graphite cathode, and silicon production; smokers and non-smokers; urine samples collected at the beginning of the first shift of the working week, post-shift on the last-but-one workday, and the beginning of the last shift of the week; France	Exposure: urinary 1-OH-PYR and 3-OH-BaP		3-OH-BaP is an essential biomarker of exposure to carcinogenic PAHs	[42]
	Seven male subjects (30–60 yearsold) working in a pre-baked electrode production plan; non-smokers; spot urine samples collected in pre- and post- shift every day of the working week; Germany	Exposure: urinary 1-OH-PYR and 3-OH-BaP	Personal air sampling	Urinary 3-OH-BaP is the most relevant biomarker for estimating carcinogenic risk in subjects exposed to complex mixtures of PAHs. It has the advantage of assessing exposure to BaP, the only PAH classified as carcinogenic to humans.	[43]
	Seven non-smoking male subjects (30–60 years old) working in a prebaked electrode production plant; urine samples collected in pre-shift and post-shift every day of the working week; France	Exposure: urinary 1- and 2-OH-NAP, 2-, 3- and 9-OH-FLU, 1-, 2-, 3-, 4- and 9-OH-PHE	Personal air sampling	1- and 2-OH-NAP, 1- and 3-OH-PHE concentrations profiles were weakly explained by occupational exposure, while 2- and 3-OH-FLU and 2-OH-PHE were strongly linked with atmospheric levels. 2-OH-FLU and 2-OH-PHE were the best biomarkers of gaseous PAH exposure	[44]
	26 subjects working in production of graphite electrodes; smokers and non-smokers; analyses carried out in post-shift spot urine samples; Germany	Exposure: urinary 1-OH-PYR, OH-PHEs, and 3-OH-BaP	Personal air sampling	A strong correlation was found between 1-OH-PYR, OH-PHEs, and 3-OH-BaP concentrations (Pearson r = 0.618–0.867, *p* < 0.001). Thus 3-OH-BaP can be regarded as a reliable and sensitive biomarker for PAHs. Poor correlation was observed between BaP in air and 3-OH-BaP for workers in the production of graphite electrodes. Because of the carcinogenic potency of BaP, 3-OH-BaP is regarded as the most relevant biomarker for risk assessment rather than 1-OH-PYR and OH-PHEs.	[45]
	19 subjects working in an aluminum production plant and 66 subjects working in a graphite electrode production plant; smokers and non-smokers; urine samples pre-shift, after the weekend and after three days of exposure (at the end of the third work day), or at the end of the work week; Slovakia	Exposure: urinary 1-OH-PYR	Stationary and personal air sampling	Strong correlation between the concentration of urinary 1-OH-PYR and concentration of pyrene or PAHs in air	[46]
	Six male subjects (30–60-years old) working in a pre-baked electrode production plan; non-smokers; urine samples collected at the beginning and at the post-shift on the last work day of the week; France	Exposure: 1-OH-PYR and 3-OH-BaP		The delay observed for maximum urinary excretion rates of 3-OH-BaP confirmed that sampling time should be performed the next morning after exposure	[41]
	26 male subjects working in production of graphite electrodes *; smokers and non-smokers; urine and blood samples collected in post-shift; Germany	Exposure: urinary 1-OH-PYR, and OH-PHEs Effect: 8-oxo-dGuo and DNA strand breaks (blood cells)	Personal air sampling	Urinary concentrations of 1-OH-PYR and OH-PHEs in relation to PAH exposures suggested additional routes of exposure at various workplaces rather than inhalation only. Significantly increased levels of 8-oxo-dGuo and DNA strand breaks when comparing all PAH exposed workers as one group to controls. The levels of DNA damage differed between the industries. The highest levels of DNA damage were found in the graphite electrode workers (more than 2-fold compared to the other groups of PAH-exposed workers). A regression model showed a weak association between air concentration of PAH and DNA strand breaks in blood cells, while no association was found between 8-oxo-Guo and PAH air exposure.	[47]
	24 male subjects working in production of graphite electrodes; smokers and non-smokers; urine samples collected in post-shift; Germany	Exposure: 1-OH-PYR, 1,6- and 1,8-OH-PYR, 1-, 2-, 3-, 4-, and 9-OH-PHE, 1,2-, 3,4-, and 9,10-OH-PHE	Personal air sampling	Association of PYR with 1-OH-PYR and 1,6- and 1,8-OH-PYR was weaker than for PHE with OH-PHEs and 1,2-OH-PHE. 1,2-OH-PHE is a new biomarker to assess occupational exposure to PHE	[48]
Converter infeed	26 workers exposed to binding pitch-containing refractories and stamping materials; smokers and non-smokers; analyses carried out in post-shift spot urine samples; Germany	Exposure: urinary 1-OH-PYR, OH-PHEs, and 3-OH-BaP	Personal air sampling	A strong correlation was found between 1-OH-PYR, OH-PHEs, and 3-OH-BaP concentrations. Thus 3-OH-BaP can be regarded as a reliable and sensitive biomarker for PAHs. No correlation was observed between BaP in air and 3-OH-BaP for workers in converter infeed. Because of the carcinogenic potency of BaP, 3-OH-BaP is regarded as the most relevant biomarker for risk assessment rather than 1-OH-PYR and OH-PHEs.	[45]
	Six male subjects exposed to PAHs during feeding converters in steel production; smokers and non-smokers; urine samples collected in post-shift; Germany	Exposure: 1-OH-PYR, 1,6- and 1,8-OH-PYR, 1-, 2-, 3-, 4-, and 9-OH-PHE, 1,2-, 3,4-, and 9,10-OH-PHE	Personal air sampling	Association of PYR with 1-OH-PYR and 1,6- and 1,8-OH-PYR was weaker than for PHE with OH-PHEs and 1,2-OH-PHE. 1,2-OH-PHE was pointed as a new biomarker to assess occupational exposure to PHE	[48]
Construction and maintenance of bituminous and asphalt roads	73 mastic asphalt workers exposed to bitumen fumes and 49 not exposed construction workers; smokers and non-smokers; pre- and post-shift urinary samples collected; Germany	Exposure: 1-OH-PYR, 1-, 2- 3-, 4-, and 9-OH-PHE	Stationary sampling	Markedly higher urinary PAH concentrations were found in a subgroup of mastic asphalt workers. Further analysis at the working place demonstrated that the cause of significantly enhanced PAH exposure during handling with bitumen under high processing temperatures was not exclusively based on the fumes of bitumen alone, but also on the contamination with coal-tar traces in the underground material as a major source of PAH exposure	[49]
	Six workers working with rolled asphalt followed by mastic asphalt one week late; smokers and non-smokers; spot urine, blood and induced sputum samples collected pre- and post-shift; Germany	Exposure: urinary 1-OH-PYR and OH-PHEs Effect: 8-Oxo-dGuo and (+)-anti-BPDE DNA adducts	Stationary and personal air sampling	Processing mastic asphalt was associated with a higher bitumen and PAH concentrations than in rolled asphalt application. However, the post-shift urinary PAH metabolite concentrations did not reflect these different exposure levels. No differences in the excretion of urinary PAH metabolites, lung function impairment or genotoxicity markers were detected comparing the two asphalt applications. Increase in 8-oxo-dGuo adducts were observed during shift while the DNA strand break levels decreased (independent of asphalt application)	[50]
	218 workers exposed to vapors and aerosols of bitumen and 96 roadside construction workers not working with asphalt (not exposed); urine and blood samples collected in pre- and post-shift; Germany	Exposure: urinary 1-, 2 + 9-, 3-, and 4-OH-PHE and 1-OH-PYR Susceptibility: The influence of 18 single-nucleotide polymorphisms (SNP) in genes coding for enzymes involved in PAH and amine metabolism regarding their impact on urinary markers (1-OH-PYR) and the sum of 1-, 2 + 9-, 3-, 4- (OH-PHE).	Personal air sampling	Significant modulation of the levels of OH-PHEs but not of 1-OH-PYR by two (GSTM1 * 1 and NAT2 * 803GG) out of 18 sequence variants of metabolizing enzymes.	[51]
	320 male workers exposed to bitumen fumes and 118 road-side construction workers (not exposed); smokers and non-smokers; spot urine and blood samples collected in pre- and post-shift; Germany	Exposure: urinary 1-OH-PYR, the sum of 1-, 2-, 3-,4-, and 9-OH-PHEs, and 1- and 2-OH-NAP Effect: (+)-anti-BPDE, 8-oxo-dGuo and DNA strand breaks (blood cells)	Personal air sampling	Increased levels of 8-oxo-dGuo adducts and DNA strand breaks in exposed workers (both pre- and post shift) compared to controls. The level of 8-oxo-dGuo was increased post- compared to pre-shift, whereas the level of DNA strand breaks was decreased post- compared to pre-shift. No difference in the level of (+)-anti-BPDE-DNA adducts between exposed and controls No association between air exposure to bitumen and biomarkers of exposure and biomarkers of effect were found. Similar no associations between PAH metabolites and DNA damage except for a weak association between 1-OH-PYR and DNA strand breaks (r_s_ = 0.18, *p* = 0.0012) were found.	[52]
	317 male bitumen-exposed workers and 117 roadside construction workers as not exposed; smokers and non-smokers; Germany	Exposure: urinary 1- and 2-OH-NAP, 1-, 2,9-, 3-, and 4-OH-PHE, 1-OH-PYR	Personal air sampling	Exposure to asphalt resulted in an additional but marginal internal PAH exposure when assessed with 1-OH-PYR and OH-PHEs in post-shift urines, whereas OH-NAP concentrations were dominated by smoking. The dose–response relation between airborne bitumen concentrations and PAH metabolites was weak	[53]
	Seven subjects working in bitumen production and asphalting plant; smokers and non-smokers; urine samples collected before work, after the weekend and after three days of exposure (at the end of the third work day), or at the end of the work week; Slovakia	Exposure: urinary 1-OH-PYR	Stationary and personal air sampling	Strong correlation between the concentration of urinary 1-OH-PYR and concentration of pyrene or PAHs in air	[46]
	91 mastic asphalt workers (bitumen-exposed group) and 42 workers from outdoor construction sites (not bitumen-exposed group); smokers and non-smokers; urine samples collected in pre- and post-shift; Germany	Exposure: urinary 1-OH-PYR, 1,6- and 1,8-OH-PYR, 1-, 2-, 3-, 4-, and 9-OH-PHE, and 1,2- and 9,10-OH-PHE	Personal air sampling	None of the PAH metabolites can be considered as a specific biomarker for bitumen exposure	[54]
	144 male workers (22–62 years) working on the paving of a new highway; smokers and non-smokers; urine samples collected in post-shift after at least three work-shifts; Italy	Exposure: urinary 2-OH-NAP and 1-OH-PYR	Stationary and personal air sampling	Higher relevance of 1-OH-PYR as compared to 2-OH-NAP for the risk assessment of hot mix asphalt exposed workers	[55]
Roofing	73 roofers and 57 subjects not occupationally exposed to PAHs; three exposure groups including soft-applied roofing using polymer-modified bitumen, hot-applied roofing using oxidized bitumen and the tearing off of old roof coatings containing coal tar; smokers and non-smokers; urine samples collected at the beginning of the week at the beginning of a shift and at the end of the week (following 3–5 days of roofing activities) either in pre- or post-shift, or 16 h after the end of a shift France	Exposure: urinary 1-OH-PYR, 3-OH-BaP, tetraolBaP, 1- and 2-OH-NAP, 1-, 2-, 3-and 9-OH-FLU and 1-, 2-,3-, 4 and 9-OH-PHE	Personal air sampling	Urinary 1-OH-PYR, 3-OH-FLU and 2-OH-PHE appeared to be the most relevant biomarkers for assessing PAH exposure in roofers due to their correlation with airborne levels of parent PAHs. They were hardly influenced by confounding factors (smoking and other environmental sources). Conversely, 1- and 2-OH-NAP should not be used for occupational exposure as they originate from many environmental sources (vehicle exhaust and tobacco)	[56]
Coke production	87 coke-oven workers; smokers and non-smokers; analyses carried out in post-shift spot urine samples; Germany	Exposure: urinary 1-OH-PYR, OH-PHEs, and 3-OH-BaP	Personal air sampling	A good correlation was found between 1-OH-PYR, OH-PHE, and 3-OH-BaP concentrations. Thus 3-OH-BaP can be regarded as a reliable and sensitive biomarker for PAHs. Statistically significant correlations are observed for workers in coking plants. Because of the carcinogenic potency of BaP, 3-OH-BaP is regarded as the more relevant biomarker for risk assessment rather than 1-OH-PYR r and OH-PHEs	[45]
	27 male coke-oven workers; smokers and non-smokers; urine samples collected post-shift; Germany	Exposure: 1-OH-PYR, 1,6- and 1,8-OH-PYR, 1-, 2-, 3-, 4-, and 9-OH-PHE, 1,2-, 3,4-, and 9,10-OH-PHE	Personal air sampling	Association of PYR with 1-OH-PYR and 1,6- and 1,8-OH-PYR was weaker than for PHE with OH-PHEs and 1,2-OH-PHE. 1,2-OH-PHE was resulted to be a new biomarker to assess occupational exposure to PHE	[48]
	37 male coke-oven workers *; smokers and non-smokers; urine and blood samples collected in post-shift; Germany	Exposure: urinary 1-OH-PYR, and OH-PHEs Effect: 8-oxo-dGuo and DNA strand breaks (blood cells)	Personal air sampling	Urinary concentrations of 1-OH-PYR and OH-PHEs in relation to PAH exposures suggested additional routes of exposure at various workplaces rather than inhalation only. Significantly increased levels of 8-oxo-dGuo and DNA strand breaks when comparing all four groups of PAH exposed workers to controls. The levels of DNA damage differed between the industries. The highest levels of DNA damage were found in the graphite electrode workers (more than 2-fold compared to the other groups of PAH exposed workers). A regression model showed a weak association between air concentration of PAH and DNA strand breaks in blood cells, while no association was found between 8-oxo-dGuo and PAH air exposure.	[47]
	104 male coke-oven workers and 49 male subjects from the general population living in the same area (controls); smokers and non-smokers; urine samples collected at the end of an 8-h work shift, after three consecutive days of work; Poland	Exposure: urinary un-metabolized PAHs (PHE, ANT, FLT, PYR, CHR, BaA, BkF, BbF, BaP, DahA, BghiP, In[c,d]P)		BaP and other carcinogenic PAHs were quantified for the first time in urine samples from both occupationally and environmentally exposed subjects, assessing exposure to specific compounds	[57]
	619 coke-oven workers (average age, 50 years old); smokers and non-smokers; urine samples collected after two non-working days before work and after the fourth day of work; Poland	Exposure: urinary 1-OH-PYR	Stationary and personal air sampling	1-OH-PYR resulted to be an effective biomarker for exposure to PAHs, due to positive correlation with air monitoring	[58]
	647 coke plant workers (18−63 years old) and 206 non-exposed subjects (18−73 years old) living in the vicinity of the coke plant but not employed therein; smokers and non-smokers; worker urine samples collected before the work shift after two non-working days and at the end of the last day of the working week; Poland	Exposure: urinary 1-OH-PYR		Urine samples of coke plant workers collected before and after the working week had higher 1-OH-PYR concentrations compared to non-occupationally exposed subjects, thus indicating that samples collected at the beginning of the working week are not suitable for assessment of the worker background exposure. Despite the fact that occupational exposure induces a much greater influence on urinary 1-OH-PYR concentrations than cigarettes smoking, the latter’s effect was still significant	[59]
Wood preservation	144 male workers (22–62 years old) handling with creosote; smokers and non-smokers; urine samples in pre- and post-shift; Germany	Exposure: urinary 1,2-OH-NAP, 1- and 2-OH-NAP, 1-NMA and 2-NMA	Stationary and personal air sampling	No significant correlations were observed between the naphthalene concentration in the air and the naphthalene metabolites, probably due to an additional uptake via the skin that may be a relevant absorption pathway in these workers. 1,2-OH-NAP and 1-NMA resulted to be specific biomonitoring parameter of naphthalene exposure	[60]
	Workers exposed to creosote and its constituents; urine samples collected before the first shift at the start of a working week that was preceded by a work-free weekend (pre-shift samples), and after three successive work days at the end of the shift (post-shift samples); Germany	Exposure: urinary 1-OH-PYR	Personal air sampling and dermal PAH exposure measurement (body Tyvek™ coveralls and split leather gloves as dermal samplers)	The results of the study confirmed the eminent extent of dermal exposure at these workplaces	[61]
Oil spills and cleanup operations	22 subjects participating in an ‘oil-on-water’ field trial in the North Sea; smokers and non-smokers; urine samples collected in pre- and post-shift; Norway	Exposure: urinary 1-OH-PYR	Personal air sampling	Urinary levels of 1-OH-PYR were within the reference range of what is considered as background level	[62]
Waste incinerator	29 workers including plant workers (incinerator operators, boiler maintenance, furnace maintenance, control panel, and waste-gas-washing operators), laboratory workers, and administration workers; Spain	Exposure: urinary 1-OH-PYR (analysis of pooled samples)		No evidence of occupational exposure to PAHs was observed	[63]
	29 workers including plant workers (incinerator operators, boiler maintenance, furnace maintenance, control panel, and waste-gas-washing operators), laboratory workers, and administration workers; Spain	Exposure: urinary 1-OH-PYR (analysis of pooled samples)		No evidence of occupational exposure to PAHs was observed	[64]
Firefighters	Four smoke diving trainers participating in three fire house simulator tests; urine samples collected before exposure, immediately after the work shift, 6 h and the next morning after exposure; Finland	Exposure: urinary 1-OH-PYR, 1-OH-NAP, and muconic acid	Stationary sampling and dermal exposure measurements	Glueless wood or gas were resulted as the safest burning material, and sinol as the safest firing liquid. Moreover, it is absolutely imperative that polystyrene foam no longer be burned	[40]
	13 male smoke diving trainers in different smoke diving simulators; urinary samples collected before exposure, immediately after the work shift, 6 h and the next morning after exposure; Finland	Exposure: urinary 1-OH-PYR and 1-OH-NAP	Stationary sampling	Dermal exposure plays a role in PAH exposure during smoke diving. In order to measure dermal exposure, a second urine sample should be taken 6 h after the exposure has ended	[65]
	153 workers including non- exposed firefighters (subjects not involved in fire combat activities within 48 h prior the urine collection) and exposed firefighters (subjects actively involved in fires combat and extinction); non-smokers; urine samples collected in post-shift; Portugal	Exposure: urinary 1-OH-NAP, 1-OH-ACE, 2-OH-FLU, 1-OH-PHE, 1-OH-PYR, and 3-OH-BaP	Personal air sampling	1-OH-NAP and 1-OH-ACE were the most abundant urinary metabolites in non-exposed and exposed firefighters. 1-OH-PYR was the less abundant biomarkers. Inclusion of other metabolites was suggested, in addition to 1-OH-PYR to better estimate occupational exposure to PAHs	[66]
	108 healthy firefighters serving at three different fire stations, smokers and non-smokers; urine samples collected post-shift between Tuesday and Thursday; Portugal	Exposure: urinary 1-OH-NAP, 1-OH-ACE, 2-OH-FLU, 1-OH-PHE, 1-OH-PYR, and 3-OH-BaP		2-OH-FLU was the most affected compound by firefighting activities; 1-OH-NAP and 1-OH-ACE presented the highest increments due to tobacco consumption	[67]
	48 firefighter workers, non-smokers; urine samples collected post-shift; Portugal	Exposure: urinary 1-OH-NAP, 1-OH-ACE, 2-OH-FLU, 1-OH-PHE, 1-OH-PYR, and 3-OH-BaP	Personal air sampling	Moderate to strong correlations were observed between PAHs and urinary OH-PAHs. In accordance with the airborne PAHs profile, urinary 1-OH-NAP and 1-OH-ACE were the predominant metabolites. Thus, total body burden of PAHs should not be based exclusively on 1-OH-PYR biomonitoring	[68]
	43 young subjects training to become firefighters **; biological measurements and samples collected 14 days before the smoke-diving course, immediately after the 3-day course exercises and 14 days subsequent to the end of the training session, Denmark Denmark	Exposure: urinary 1-OH-PYR Effect: Cardiovascular effects (microvascular function, heart rate variability)	Stationary and personal air sampling	Fire extinction exercises were associated with increased urinary 1-OH-PYR levels, decreased microvascular function and altered heart rate variability. However, the association between urinary 1-OH-PYR excretion and cardiovascular effects was not statistically significant in models that included smoke exposure as categorical variable	[69]
	53 young subjects training to become firefighters; biological samples collected 14 days before the smoke-diving course, immediately after the 3-day course exercises and 14 days subsequent to the end of the training session; Denmark	Exposure: urinary 1-OH-PYR Effect: Inflammatory markers in plasma (ICAM-1, VCAM-1, SAA, CRP, IL-6, IL-8. DNA damage in peripheral blood mononuclear cells (DNA strand breaks and Fpg-sensitive sites). Lung function	Dermal wiping of the neck	Increased urinary excretion of 1-OH-PYR after the firefighting exercise compared with the two control measurements performed 2 weeks before and 2 weeks after the firefighting course, respectively. The level of DNA strand breaks was strongly associated with total PAH and pyrene levels on skin and urinary excretion of 1-OH-PYR.	[70]
	22 male subjects from 3 consecutive 24 h work shift days (39–59 years old); urine samples collected pre- and post-shift and 24 h after the shift; Denmark	Exposure: urinary 1-OH-PYR Effect: Inflammatory markers in plasma (ICAM-1, VCAM-1, SAA, CRP, IL-6, IL-8. DNA damage in PBMC (DNA strand breaks, Fpg-sensitive sites), lung function;	Dermal wiping of the neck	No increase in the PAH levels on the skin (back of the neck) nor 1-OH-PYR concentrations in urine was observed. The work shift was not associated with increased levels of genotoxicity or decreased lung function, while increased levels of VCAM-1 was observed.	[6]
	20 students (19–29 years old) at the Swedish Civil Contingencies Agency for prospective firefighters; non-smokers; urine samples collected pre-shift and at 6 and 20 h after exposure; Sweden	Exposure: urinary 1- and 2-OH-NAP, 1-OH-ACE, 2- and 9-OH-FLU, 9-OH-PHE, 1-OH-PYR, 3-OH-BaP	Stationary sampling inside the smoke container, air sampling inside the instructor’s Jacket, skin wipe sampling	Median urinary 1-OH-PYR levels were significantly increased after 6 h and 20 h as compared to baseline levels. The other OH-PAHs showed significantly increased levels after 6 h but not after 20 h, indicating a faster overall biotransformation. 1-OH-PYR showed the strongest correlation to skin-deposited PAHs and therefore it is suggested that this is a better indicator of exposure during smoke diving exercises when wood is used as fuel	[71]
	Six firefighting instructors (20–41 years old) exposed to combustion products in a compartment fire behavior training unit; non-smoker; repeated collection of urine samples before and immediately after, as well as 1, 3, 6, 9, 11, and 18 h after each training session (five training sessions, the time interval between two consecutive training sessions of the same test person being at least six days); Germany	Exposure: urinary 1- and 2-OH-NAP, 2-, 3-and 9-OH-FLU, 1-, 2-,3- and 4-OH-PHE, and 1-OH-PYR		A significant effect of the training sessions on the time course of internal exposure was found. OH-PAHs concentration increased at the latest 3 h after end of training. Due to the use of self-containing breathing apparatuses, dermal absorption is assumed as major exposure route	[72]
Policemen	374 workers; smokers and non-smokers; urine samples collected after 5 working days, post-shift; Italy	Exposure: urinary 1-OH-PYR Effect: blood pressure		Occupational exposure to PAHs may be able to significantly influence the blood pressure probably acting on the autonomic nervous system. Inverse correlation between 1-OH-PYR and blood pressure	[73]
Bus drivers	50 bus drivers and 50 controls spending >90% of daily time indoors, non-smokers; spot urine samples and blood collected post-shift; Czech Republic	Effect: 8-oxo-dG (urine)	Personal air sampling (once in each season)	Bus drivers were exposed to significantly higher levels of PAHs in winter, while in the other two seasons the exposure of controls was unexpectedly higher than that of bus drivers. 8-oxo-dG levels were higher in bus drivers than in controls in all seasons	[74]
Air force personnel	79 employees at an air force military base grouped by exposure based on job-function; non-smokers; first morning spot urine samples collected; Denmark	Exposure: urinary 1- and 2-OH-NAP, 2-OH-FLU, 1-, 2 + 3-, and 4-OH-PHE, and 1-OH-PYR Effect: Plasma level of acute phase response (SAA and CRP); genotoxicity (DNA strand breaks in PBMCs and micronuclei frequency in blood reticulocytes); lung function	PAH exposure assessed by silicon bands used as samplers; dermal exposure assessed by neck and hand palm wiping	No difference in exposure levels of total PAHs and OH-PAHs between exposure groups/job functions. No effects for biomarkers of systemic inflammation, genotoxicity; or lung function	[75]
Maintenance of parks, gardens, and reserves	48 green space workers (20–55 years old) exposed to traffic-related air pollution; smokers or non-smoker; urine sample collected at the end of the fourth day follow-up; Belgium	Exposure: urinary 1- and 2-OH-NAP, 1-OH-PYR Effect: Airway inflammation (Exhaled NO); oxidative stress (8-OHdG in urine)	Personal air sampling (black carbon exposure)	With the exception of 2-OH-NAP, PAH biomarkers were measured below their detection limits in 70% (1-OH-PYR) and half of the samples (1-OH-NAP). Therefore, PAH exposure was not tested for potential correlations to health conditions.	[76]
Healthcare workers	66 dermatology nurses involved in the topical application of coal tar ointment; smokers and non-smokers, The Netherlands	Exposure: urinary 1-OH-PYR	Personal air and dermal sampling	Use of gloves reduced the excretion of 1-OH-PYR by 51.5%	[77]
	15 surgeons, scrub assistants, and circulation nurses exposed to surgical smoke in an operating room; pre-, mid-, and end-shift urine samples collected (mid- and end-shift urinary sampling performed 30 min to 2 h after the cessation of smoke production); Belgium	Exposure: urinary 1-OH-PYR	Stationary and personal air sampling	1-OH-PYR generally measured below its detection limit. No correlation was found between naphthalene in air and urinary 1-OH-NAP	[78]
Workers in restaurants	96 employees in restaurants, smokers and non-smokers; Portugal	Effect: Lung function, TAS (plasma), 8-OHdG (serum) and proteomics analysis of plasma	Stationary sampling	Increased levels of 8-OHdG in secondhand tobacco smoke exposed workers compared to non-exposed workers (regardless of smoking status). Proteomics analysis showed 9 differentially expressed proteins in plasma of second-hand tobacco smoke exposed non-smokers. Among these two acute phase proteins (ITIH4) and CP were expressed the most	[79]
	18 grill workers; non-smokers; urine samples collected at the end of the working day over a complete working week, including the resting days; Portugal	Exposure: urinary 1-OH-NAP + 1-OH-ACE, 2-OH-FLU, 1-OH-PHE, 1-OH-PYR, and 3-OH-BaP		OH-PAH concentrations were significantly increased during subjects’ working period compared with the following resting days, being 1-OH-NAP + 1-OH-ACE and 2-OH-FLU the compounds with the highest increments and 1-OH-PYR the lowest increments	[80]

* Part of a larger cross-sectional study [47] with 171 workers in total [coke oven (n = 37), refractory (n = 96), graphite electrode (n = 26), converter workers (n = 12); construction workers (n = 48) served as control group]; ** Subgroup of the 53 participants in [69]; Abbreviations: 8-oxo-dG: 8-oxodeoxyguanosine; 8-oxo-dGuo: 8-oxo-7,8-dihydro-2′-deoxyguanosine; 8-OHdG: 8-hydroxyguanosine; VOC: volatile organic compounds; (+)-anti-BPDE: (+)-anti-benzo(a)pyrene-7,8-diol-9,10-epoxide; 1-OH-PYR: 1-hydroxypyrene, ∑OH-PHE(1): 1-, 2 + 9-, 3-, and 4-hydroxyphenanthrenes; ∑OH-PHE(2): 1-, 2-, 3-, 4-, and 9-hydroxyphenanthrenes; ∑OH-NAP: 1- and 2-hydroxynaphtalene; TAS: total antioxidant status; ITIH4: inter-alpha-trypsin inhibitor heavy chain 4; CP: ceruloplasmin; ICAM-1: intercellular cell adhesion molecule 1: VCAM-1: vascular cell adhesion molecule 1; SAA: serum amyloid protein; CRP: C-reactive protein; IL-6: interleukin-6; IL-8: interleukin-8, PBMC: peripheral blood mononuclear cells.

**Table 5:** Summary of reported effects related to genotoxicity/oxidative stress.

Study Type	8-Oxo-dGuo	8-OHdG	8-Oxo-dG	(+)-Anti-BPDE	DNA Strand Breaks	Fpg-Sensitive Sites	Micronuclei	Ref.
Cross-sectional	↑ (blood)				↑ (blood)			[47]
Cross-sectional/cross-shift	↑ (blood)			=	↑ (blood)			[52]
Longitudinal study	↑ during shift (blood)			=				[50]
Cross-sectional			↑ (urine)					[74]
Cross-shift (participants are their own controls)					=(PBMC)	↓ (PBMC)		[6]
Longitudinal (participants are their own controls)					= (PBMC)	↑ (PBMC)		[70]
Cross-sectional					= (PBMC)		= (PBR)	[75]
Second-hand tobacco smoke exposed workers compared to non-exposed workers *		↑						[79]
Cross-sectional		**						[76]

8-oxo-dGuo: 8-oxo-7,8-dihydro-2′ -deoxyguanosine; 8-Oxo-dG: 8-oxodeoxyguanosine; 8-OHdG: 8-hydroxyguanosine; PBMC: peripheral blood mononuclear cells; PBR: peripheral blood reticulocytes. * 8-OHdG measured in blood serum; ** The level of 8-OHdG is reported for the whole group of 48 workers. No comparison is provided; ↑—increased; ↓—decreased.; = no effect.

**Table 6:** Summary of reported effects related to cardiovascular effects.

Study Type	Blood Pressure	Microvascular Function	Heart Rate Variability	SAA	CRP	IL-6	IL-8	I-CAM	V-CAM	ITIH4 and CP	Ref.
Second-hand tobacco smoke exposed workers compared to non-exposed workers										↑	[79]
No control group: “*when assessing the correlation between the dose of urinary 1-OH-PYR and BP in standing and supine positions, the “control group” was inherent in the sample*”	Inverse correlation between 1-OH-PYR and blood pressure										[73]
Human exposure study, where the participants were studied in three exposure scenarios, serving as their own controls.		↓	↓ (SDNN, pNN50 and RMSSD, HF) ↑ (LF, LF/HF ratio)								[69]
				=	=	<LOD	<LOD	=	=		[70]
Workshift, comparison of pre-shift with post-shift				=	=	<LOD	<LOD	=	↑		[6]
Cross-sectional				=	=						[75]

LOD: level of detection; HF: high-frequency components; LF: low-frequency components. ↑—increased, ↓—decreased.

## Data Availability

Not applicable.

## Supplementary Materials

The following supporting information can be downloaded at: https://www.mdpi.com/article/10.3390/toxics10080480/s1, Table S1: Kinetic characteristics (tmax: time to maximal value after administration; t1/2: urinary elimination half-life) of urinary PAH metabolite elimination after oral, inhalation and dermal exposure; Table S2: Results of the Lakind scoring (excluding effect biomarkers) of the publications considering the aim of this study. Each category was scored out of 3, the lower scores indicating the better quality.
