# Supplementary material for: The Use of Human Biomonitoring to Assess Occupational Exposure to PAHs in Europe: A Comprehensive Review
Source: Toxics. 2022 Aug 17;10(8):480. doi: 10.3390/toxics10080480 (PMC9414426; doi:10.3390/toxics10080480)
Supplement: Supplementary file 1 [file toxics-10-00480-s001.zip › toxics-1817072-supplementary.pdf]

Table S1: Kinetic characteristics (t_{max}: time to maximal value after administration; $t_{1/2}$: urinary elimination half-life) of urinary PAH metabolite elimination after oral, inhalation and dermal exposure

PAHs metabolite	Oral ingestion[1,2]		Inhalation exposure[3,4]	Dermal exposure[5]	
	**t_{max} [h]**	$t_{1/2}$ [h]	$t_{1/2}$ [h]	t_{max} [h]	$t_{1/2}$ [h]
1-naphthol	3.1[1]	4.3[1]; 3.4[2]	-	-	-
2-naphthol	5.8[1]	2.5[1]; 2.4[2]	9.4[3]	-	-
1-hydroxyfluorene(1-OHFlu)	-	-	5.5[3]	-	-
2-hydroxyfluorene (2-OHFlu)	3.9[1]	2.9[1]; 2.6[2]	4.1[3]	-	-
3-hydroxyfluorene (3-OHFlu)	3.9[1]	6.1[1]; 7.0[2]	8.2[3]	-	-
9-hydroxyfluorene (9-OHFlu)	3.8[1]	3.1[1]; 1.7[2]	-	-	-
1-hydroxyphenanthrene (1-OHPh)	5.3[1]	5.1[1]; 3.1[2]	-	-	-
2-hydroxyphenanthrene (2-OHPh)	4.1[1]	3.9[1]; 3.7[2]	-	-	-
3-hydroxyphenanthrene (3-OHPh)	5.1[1]	4.1[1]; 2.6[2]	-	-	-
4-hydroxyphenanthrene (4-OHPh)	3.8[1]	3.5[1]; 2.9[2]	-	-	-
1-hydroxypyrene (1-OHP)	5.5[1]	3.9[1]; 4.4[2]	6.0[3]; 9.8[4]	12.5	11.8

[1] Dietary exposure to a high PAHs-contaminated lunch (barbecued chicken). Data from [17] (mean values for t_{max}, medians for $t_{1/2}$)
[2] Dietary exposure to a high PAHs-contaminated lunch (traditional smoked salmon). Data from [18] (median values).
[3] Inhalative exposure by cigarette smoking. Data from St Helen et al. 2012. DOI: 10.1021/tx300043k (mean values)
[4] Inhalative exposure to workplace air of an aluminium plant. Data from [7] (one- compartment model)
[5] Dermal exposure of volunteers to a coal tar-base shampoo or to 100 µl creosote. Data from [26] (mean values)

Table S2: Results of the Laking scoring (excluding effect biomarkers) of the publications considering the aim of this study. Each category was scored out of 3, the lower scores indicating the better quality.

Total Score	Study Participants Score	Chemicals under Investigation Score	Exposure Biomarker and Matrix Score	Exposure Biomarker Specificity	Technique Quality Check Score	Method Quality Check Score	QA Score	Matrix Adjustment Score	Ref.
10	2	2	1	1	1	1	1	1	[41]
11	1	2	1	1	1	1	2	2	[42]
11	1	2	1	1	1	1	2	2	[57]
11	1	2	2	1	1	1	1	2	[47]
11	1	2	2	1	1	1	1	2	[75]
11	1	1	1	1	1	1	3	2	[56]
12	**2**	**2**	**1**	1	**1**	1	2	2	[16]
12	**2**	**2**	**2**	1	**1**	1	1	2	[44]
12	1	2	1	1	1	1	3	2	[45]
12	1	1	2	1	1	1	3	2	[58]
12	1	2	1	1	1	1	3	2	[66]
12	1	2	1	1	1	2	3	1	[77]
12	1	2	2	1	1	1	2	2	[55]
12	2	2	2	1	1	1	1	2	[60]
13	2	2	1	1	1	1	3	2	[67]
13	1	2	2	1	1	1	3	2	[73]
13	1	1	2	1	1	2	3	2	[46]
13	1	2	2	1	1	1	3	2	[51]

13	1	2	2	1	1	1	3	2	[63]
13	1	2	2	1	1	1	3	2	[48]
13	1	2	2	1	1	1	3	2	[53]
13	1	2	2	1	1	1	3	2	[68]
13	1	2	2	1	1	1	3	2	[54]
13	1	2	2	2	1	1	2	2	[6]
13	1	2	2	2	1	1	2	2	[76]
13	1	1	2	2	1	2	2	2	[61]
13	1	2	2	2	1	1	2	2	[70]
14	3	2	1	1	1	1	3	2	[80]
14	2	1	2	2	1	1	3	2	[78]
14	1	2	2	2	1	1	3	2	[59]
14	3	1	2	1	1	1	3	2	[71]
14	1	2	2	2	1	1	3	2	[69]
15	1	3	2	1	1	2	3	2	[62]
15	1	2	2	1	1	3	3	2	[64]
15	1	2	2	1	1	3	3	2	[52]
15	1	2	3	2	1	2	3	1	[79]
15	2	2	2	2	1	1	3	2	[72]
16	1	2	2	1	2	3	3	2	[49]
17	2	2	2	1	2	3	3	2	[50]
17	3	2	2	1	1	3	3	2	[65]
17	1	1	3	2	2	3	3	2	[74]
18	3	2	2	1	1	3	3	3	[40]
